# Widely Targeted Metabolomics Revealed the Metabolic Basis of Physiological Function and Flavor of Natto

**DOI:** 10.3390/metabo14120663

**Published:** 2024-12-01

**Authors:** Xiaolong Yin, Xiaona Wang, Lili Xu, Jianzhi Zhao, Can Li, Jianqiang Lin

**Affiliations:** 1 School of Bioengineering, Qilu University of Technology (Shandong Academy of Sciences), Jinan 250353, China; 10431221235@qlu.edu.cn (X.Y.); xulili@qlu.edu.cn (L.X.); zhanjianzhi@qlu.edu.cn (J.Z.); 2 Shandong Freda Biotech Co., Ltd., Jinan 250101, China; frdsw@biofreda.com; 3 State Key Laboratory of Microbial Technology, Shandong University, Qingdao 266200, China

**Keywords:** natto, metabolite profiling, UHPLC-MS/MS, KEGG

## Abstract

**Background:** Natto is a fermented product derived from soybeans through the action of Bacillus subtilis natto, possessing various pharmacological and health-promoting properties. However, due to the absence of large-scale and systematic investigations into its metabolite profile, the mechanisms governing the biological functions and flavor characteristics of natto remain incompletely elucidated. **Methods:** In this study, a comprehensive, widely targeted metabolome analysis was conducted using UHPLC-MS/MS to compare soybeans and natto. **Results:** A total of 569 metabolites were identified, of which 160 exhibited differential expression between natto and soybeans, including 28 amino acids and their derivatives, 19 flavonoids, 18 alkaloids, and 10 nucleotides and their derivatives. Pathway enrichment analysis further demonstrated significant differences in the metabolic pathways between natto and soybeans, with these 160 differentially expressed metabolites primarily distributed across 40 metabolic pathways. KEGG pathway enrichment analysis of natto metabolites revealed that the majority of these mapped to three key metabolic pathways. Variations in the content of flavonoids and alkaloids, as well as changes in amino acid and saccharide composition and abundance, were found to collectively contribute to the distinctive flavor and biological functionality of natto. **Conclusions:** This study lays the foundation for future efforts to enhance the quality of natto.

## 1. Introduction

Natto is a traditional fermented food produced using *Bacillus subtilis natto* or *Bacillus subtilis*, with soybeans serving as the primary raw material. This highly nutritious and health-enhancing food has a rich historical background and is widely consumed not only in Japan but also in other Asian countries and various regions worldwide [1]. Natto represents an economical yet extraordinarily nutrient-rich product, manufactured through a simple and straightforward process. In Japan, in particular, it is esteemed as a culinary key to longevity, with a consumption tradition spanning several centuries [2]. This cost-effective yet highly nutritious product is created via a simple methodology, historically carried out by individual households for their own use. The production process involves soaking soybeans, cooking them until tender, draining and cooling them to 40 °C, inoculating them with a *B. subtilis natto* suspension, and fermenting at 40–43 °C for 12–20 h [3,4]. High-quality natto is distinguished by its characteristic white mucilaginous layer, unique aroma, soft and agreeable texture, a light golden color, and the ability to form silky, sticky threads when manipulated with chopsticks [2].

Natto not only serves as an abundant source of readily assimilable amino acids, organic acids, and oligosaccharides but also boasts a wealth of bioactive compounds, including natto kinase, superoxide dismutase, isoflavones, saponins, and vitamins [5,6,7]. Revered historically as both a culinary staple and a medicinal agent, natto has become the focus of extensive research, revealing its multifaceted pharmacological and health-promoting properties. These include thrombolytic, anti-neoplastic, antihypertensive, lipid-lowering, antioxidant, anti-osteoporotic, and pro-coagulant effects [8,9,10,11]. Regular consumption of natto is correlated with improved overall health and disease prevention, such as the reinforcement of the immune system (inhibits infection) [12], the maintenance of cardiovascular health [13], and the support of digestive functions [14]. However, the pungent, ammonia-like odor characteristic of traditional natto has somewhat impeded its global acceptance and market expansion [15].

Previous studies have illuminated the roles of isovaleric and isobutyric acids in shaping natto’s flavor profile, as well as the contributions of flavonoids, vitamin K, and other metabolites to its physiological benefits [16,17,18]. While these investigations have yielded valuable insights, they have predominantly concentrated on specific metabolite classes, providing only partial explanations for natto’s distinctive characteristics. Consequently, a comprehensive analysis involving large-scale identification and quantification of metabolites is essential to fully elucidate the contributions of various metabolite classes.

Ultra-high-performance liquid chromatography–tandem mass spectrometry (UHPLC-MS/MS)-based widely targeted metabolome analysis presents a swift and dependable method for detecting a broad spectrum of plant metabolites [19,20]. To achieve a more thorough understanding of natto’s flavor profile and functional components, we conducted UHPLC-MS/MS analysis to identify and quantify metabolites, including sugars, isoflavones, and amino acids, in both soybeans and natto. Our findings offer novel insights into the taste and bioactive compounds present in natto, providing a robust theoretical foundation for enhancing its flavor profile.

## 2. Materials and Methods

### 2.1. Fermentation of Soybeans

The clones of *Bacillus subtilis natto* X-16 were cultured in a shaking flask at a temperature of 37 °C and an agitation speed of 200 rpm for a duration of 18 h. Following incubation, the culture suspension was transferred into centrifuge tubes and subjected to centrifugation at 4 °C and 5000 rpm for 3 min to harvest the bacterial pellet. The bacterial pellet was subsequently washed three times with distilled water. Soybeans of uniform size and intact seed coats were carefully selected for the experiment. A total of 500 g of soybeans was accurately weighed and soaked in 1000 mL of water for 12 h. After thorough washing, the soybeans were placed in a sterilized container and autoclaved at 115 °C for 30 min to ensure sterilization and complete cooking. Upon cooling, the sterilized and cooked soybeans were transferred into a sterile fermentation vessel. The purified bacterial pellet was resuspended in distilled water and uniformly inoculated onto the soybeans. Following inoculation, the fermentation container was placed in an incubator maintained at 37 °C for a period of 48 h [21].

### 2.2. Sample Preparation and Metabolite Extraction

Both laboratory-fermented natto and its pre-fermentation soybean counterpart were subjected to lyophilization, with three replicate samples prepared for each group. The lyophilized samples were then pulverized using a mechanical mill for a duration of 120 s at a frequency of 60 Hz. Precisely 100 mg of each sample was accurately weighed and transferred into an Eppendorf tube, after which 1.5 mL of extraction medium (a pre-chilled 3:1 methanol-water solution at −40 °C) was added. The samples were homogenized at 40 Hz for 240 s and subsequently sonicated for 15 min in an ice bath following a brief 30 s vortex. The samples were then allowed to extract overnight at 4 °C on a shaking platform, followed by centrifugation at 12,000 rpm for 15 min at 4 °C. The resulting supernatant was carefully filtered through a 0.22 μm microporous membrane, diluted tenfold with a 3:1 methanol-water solution containing an internal standard, vortexed for 30 s, and transferred into a 2 mL glass vial for subsequent UHPLC-MS analysis [22,23].

### 2.3. Quality Control and UHPLC-MS Analysis

To ensure the reproducibility of mass spectrometric analyses, quality assurance (QA) samples were prepared by blending equal proportions of soybean and natto extracts. A composite QA sample was created by combining 100 μL aliquots from each individual sample for subsequent analysis. Chromatographic separation was achieved using an EXIONLC System with ultra-high-performance liquid chromatography (UHPLC) (Sciex, USA). The mobile phases were composed of 0.1% formic acid in water (A) and acetonitrile (B). The analytical column was maintained at 40 °C, while the auto-sampler was held at 4 °C. Sample introduction was accomplished with a 2 μL injection volume. Mass spectrometric detection was performed using a Sciex QTrap 6500+ instrument (Sciex, Framingham, MA, USA). The ion source parameters were finely tuned as follows: IonSpray Voltage was set to +5500/−4500 V, Curtain Gas pressure at 35 psi, Source Temperature at 400 °C, Ion Source Gas 1 and 2 pressures at 60 psi each, and Declustering Potential (DP) at ±100 V.

### 2.4. MS Data and Statistical Analysis

For the acquisition and analysis of MRM data, Analyst Work Station Software (Version 1.6.3, Sciex, USA) was employed. The MS raw data files (.wiff) were converted to TXT format utilizing MS Converter. Peak detection and annotation were performed with a custom R program and database [24,25,26]. Identification of metabolites and structural analysis of primary and secondary spectral data were carried out using the BIOTREE PWT database, provided by Shanghai BIOTREE Biological Technology Co., Ltd. (Shanghai, China).

Statistical analyses were conducted using R version 4.0.5 (https://cran.r-project.org/src/base/R-4/ (accessed on 10 September 2024)). Principal component analysis (PCA) was performed with the prcomp function, while supervised multiple regression orthogonal partial least-squares discriminant analysis (OPLS-DA) was executed using the ropls package (v1.19.8). To validate the model, a 200-fold permutation test was conducted. Differential metabolite accumulation was determined based on fold change criteria (≥2.0 for upregulation and ≤0.500 for downregulation) in natto compared to soybean, followed by screening using variable importance in projection (VIP) threshold (VIP ≥ 1.0) derived from the OPLS-DA model.

The statistical significance of differences in metabolite abundance was assessed using a two-tailed Student’s *t*-test (*p* = 0.05). Annotated metabolites were subsequently mapped to the Kyoto Encyclopedia of Genes and Genomes (KEGG) pathway database (http://www.kegg.jp/kegg/pathway.html (accessed on 10 September 2024)) for pathway association. MetaboAnalyst (http://www.metaboanalyst.ca/ (accessed on 10 September 2024)) were used for pathway enrichment analysis. Pathways with Bonferroni-corrected *p*-values ≤ 0.05 were considered significantly enriched.

## 3. Results

### 3.1. Widely Targeted Metabolic Analysis of Natto

**Figure 1:**
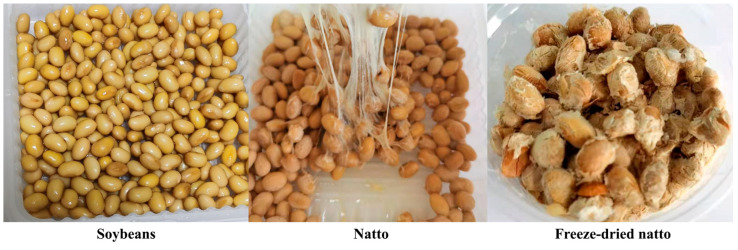
Morphology of soybeans (before fermentation), natto (fermentation completed), and freeze-dried natto (vacuum freezing after fermentation).

After fermentation, the natto showed a long stretch, indicating successful fermentation, and freeze-dry it and send it for sample testing (Figure 1). To elucidate the complex composition of flavor constituents and bioactive compounds in natto and soybean, a comprehensive metabolomic analysis was performed using ultra-high-performance liquid chromatography coupled with mass spectrometry (UHPLC-MS/MS). The study generated an extensive catalog of 569 unique metabolites, including a wide range of compounds with potential health benefits and sensory attributes. Notably, 70 flavonoids, 8 carbohydrates, and 60 amino acids were identified as potential contributors to the health-promoting properties of natto. Additionally, the analysis detected 27 nucleotides and their derivatives, 21 organic acids, 76 alkaloids, and 45 phenolic compounds, which may significantly influence the distinctive taste profile of this fermented soybean product (see Supplementary Table S1 for complete data).

### 3.2. Multivariate Analysis of Identified Metabolites

**Figure 2:**
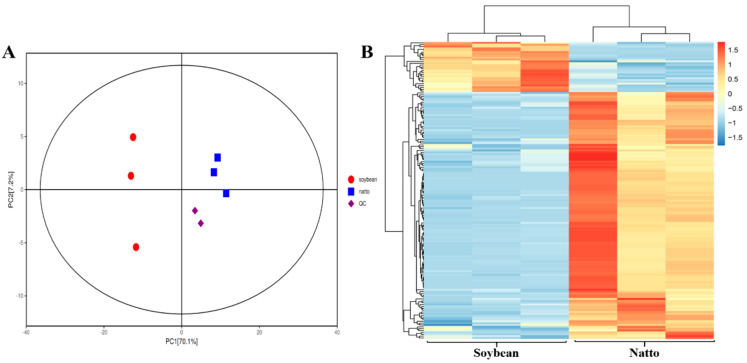
Difference in metabolite concentration in soybean and natto. (**A**) PCA of soybean and natto metabolites. Equal volumes of soybean and natto samples were mixed as a control. (**B**) Cluster analysis of soybean and natto metabolites. Colors indicate the level of accumulation of each metabolite (blue: lower concentrations; red: higher concentrations). Z-score represents a deviation from the mean in standard deviation units.

Principal component analysis (PCA) was conducted on the 569 identified metabolites. The PCA clearly discriminated between the two food products and the quality control (QC) samples with a statistical significance of 0.01 (Figure 2A). To minimize the impact of quantitative variations on pattern recognition, a logarithmic transformation (base 10) was applied to the peak areas of each metabolite, followed by hierarchical cluster analysis. This subsequent analysis identified two distinct clusters corresponding to soybean and natto, respectively (Figure 2B). Consequently, the integration of PCA and cluster analysis suggested that these two cultivars exhibited significantly different metabolite profiles.

**Figure 3:**
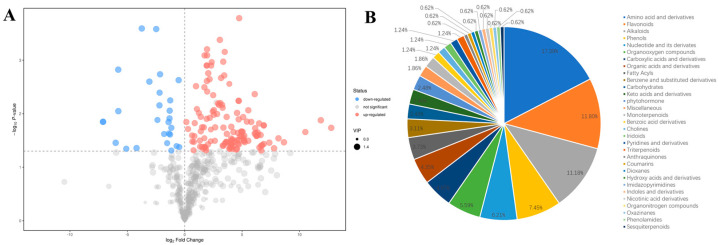
Differentially accumulated metabolites between soybean and natto. (**A**) Volcano plot of 569 metabolites identified. Differential metabolites were defined as metabolites with a fold change ≥ 2.0 or ≤0.5 in natto compared to soybeans. A threshold of VIP ≥ 1.0 was considered to separate differential metabolites from non-differential metabolites. (**B**) Biochemical categories of differential metabolites between natto and soybean.

To discern the differential metabolites between soybean and natto, a selection criterion of fold change ≥ 2.0 (upregulated) or ≤0.5 (downregulated) in natto compared to soybean was employed. Simultaneously, flavonoids were screened using variable importance in projection (VIP) value (VIP ≥ 1.0) derived from the OPLS-DA model, in conjunction with a *p*-value (*p* ≤ 0.05) obtained from Student’s *t*-test. This thorough analysis resulted in the identification of 160 differential metabolites between the two samples. Among these, 27 metabolites were found to be downregulated, while 132 metabolites were upregulated in natto relative to soybean (Figure 3A). These 160 metabolites were categorized into more than 30 distinct classes (Figure 3B), with the predominant categories including alkaloids, flavonoids, amino acids, phenols, nucleotides and their derivatives, as well as organooxygen compounds (Table S1).

### 3.3. KEGG Classification and Enrichment Analysis of Differential Metabolites

**Figure 4:**
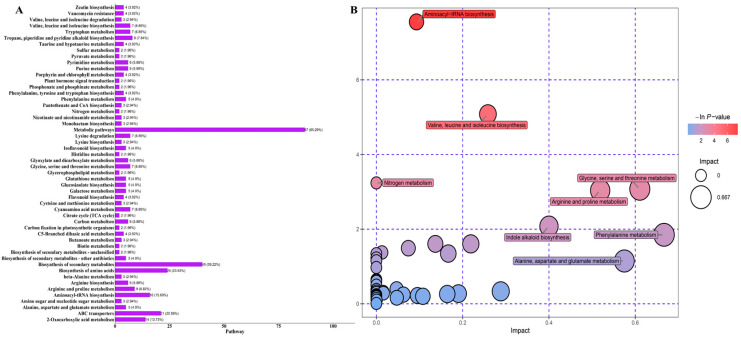
KEGG classification of differentially accumulated metabolites. (**A**) KEGG category: metabolism. (**B**) Metabolome view maps of metabolic pathways in natto and soybean.

We conducted a cross-referencing analysis of the 160 identified metabolites with the KEGG database to discern their associated pathways. As expected, the majority of these metabolites were primarily associated with the categories of ‘metabolism’ and ‘biosynthesis of secondary metabolites’ (Figure 4A, Table S2). To elucidate the metabolic variations between the two cultivars, we performed an extensive KEGG pathway enrichment analysis. The results indicated that the 160 significantly altered metabolites were mainly distributed across 40 unique metabolic pathways. Notably, the eight pathways harboring the highest number of differentially expressed metabolites were aminoacyl-tRNA biosynthesis; valine, leucine, and isoleucine biosynthesis; nitrogen metabolism; glycine, serine, and threonine metabolism; arginine and proline metabolism; indole alkaloid biosynthesis; phenylalanine metabolism; and alanine, aspartate, and glutamate metabolism (Figure 4B).

### 3.4. Differences in the Contents of Isoflavones, Purines, Saccharides, and Alkaloids in Natto and Soybean

The study detected 70 unique flavonoids in both soybeans and natto, with a significant elevation in isoflavone levels in natto (Table S1), which represent the primary flavonoid constituents. Specifically, the concentrations of genistein and glycitein in natto were 16.4 and 11.4 times greater, respectively, than those found in soybeans. This observation underscores the potential for enhancing the bioactive isoflavone content in food, marking it as a crucial area for further research. Furthermore, the study identified 27 nucleotides and their derivatives in both natto and soybeans (Table S1), with 10 showing differential accumulation and notable quantitative differences (Student’s *t*-test, *p* < 0.05). Guanosine (fold change = 287) and adenine (fold change = 118) exhibited the highest concentrations and were significantly more abundant in natto compared to soybeans. Additionally, 45 amino acids were identified (Table S1), of which 26 displayed differential accumulation. Significant variations in the levels of L-lysine, L-ornithine, 5-aminovaleric acid, and isoleucine were noted between natto and soybeans (Student’s *t*-test, *p* < 0.05).

The study also identified 19 different sugars, with D-xylulose (fold change = 220), D-glucose 6-phosphate (fold change = 26), and glucosamine (fold change = 16) showing markedly higher concentrations in natto than in soybeans. However, the absence of high-sweetness sugars like fructose and sucrose negatively impacts natto’s palatability. Moreover, 76 types of alkaloids were detected, with 68 generally upregulated, particularly the 137-fold increase in methyl anthranilate content in natto compared to soybeans, which may contribute to its complex flavor profile. In addition to flavonoids, purines, amino acids, and alkaloids, substantial amounts of benzocaine and strictosidine were found in natto. Benzocaine, a renowned anesthetic, and strictosidine, a pivotal intermediate in monoterpene indole alkaloid biosynthesis, hint at potential applications in medical anesthetics. Meanwhile, the strong antioxidant deoxyelephantopin (fold change = 105), which has immunomodulatory and anticancer effects, was also detected in natto.

## 4. Conclusions and Discussion

Comprehensive metabolite profiling analyses utilizing MS/MS data have been effectively applied to the extensive characterization of metabolites and comparative metabolomics in various important plant species [27]. Previous research on natto has primarily focused on the identification of specific metabolites associated with distinct metabolic pathways. To date, a systematic exploration of the overall differences in natto’s metabolic profiles has remained unexplored. In this study, we employed UHPLC-MS/MS-based comprehensive metabolomics to delineate the differences between natto and soybean metabonomics. A total of 569 metabolites were identified, of which 160 showed differential accumulation between natto and soybean. The accumulation patterns of these metabolites provide foundational data and insights for further elucidating the biochemical mechanisms underlying natto’s physiological functions.

It is widely recognized that natto contains vitamin K, nattokinase, and probiotics, imparting numerous physiological benefits. Our study further revealed that natto contains a significant amount of free flavonoids, notably genistein and glycine glycosides, present at 16.4 and 11.4 times the levels found in soybeans, respectively. Extensive research has shown that the biological efficacy of isoflavones is mainly attributed to their aglycones, such as daidzein and genistein, rather than their glycosides. Isoflavone aglycones have been reported to exhibit superior absorption rates and quantities in the human body, thereby enhancing their physiological benefits. Consequently, increasing the concentration of bioactive isoflavones in food has become a prominent research focus in recent years [28,29]. The isoflavone biosynthesis pathway suggests a significant upregulation of β-glucosidase activity [30] during natto fermentation, potentially explaining the elevated levels of free flavonoids in natto (Figure S1).

Meanwhile, we have discovered that the concentration of deoxyelephantopin in natto is 105 times higher than in soybeans. Deoxyelephantopin possesses potent antioxidant capabilities, effectively scavenging free radicals and safeguarding cells from oxidative stress-induced damage. It also exhibits antiviral properties, particularly against influenza and herpes simplex viruses. Moreover, deoxyelephantopin demonstrates inhibitory effects on certain types of cancer cells, such as those associated with breast, liver, and lung cancers. Its anticancer potential is further evidenced by its ability to induce apoptosis, suppress cell proliferation, and inhibit metastasis [31]. Consequently, natto exhibits a variety of biological functions, including anti-atherosclerotic, anti-inflammatory, antitumor, and antioxidant attributes.

In the human body, purines are metabolized into uric acid, serving as an antioxidant and mitigating damage caused by reactive oxygen species. However, frequent and excessive consumption of purine-rich foods has been associated with elevated serum uric acid levels, potentially leading to gout and increasing the risk of cardiovascular disease, kidney disease, and metabolic syndrome. Japanese dietary guidelines recommend a daily purine intake not exceeding 400 mg to prevent gout and hyperuricemia [32]. However, due to natto’s high content of guanine nucleosides and adenine, frequent and substantial consumption may elevate serum uric acid levels, potentially triggering gout and serving as a risk factor for cardiovascular disease, renal disorders, and metabolic syndrome. Therefore, utilizing the fundamental data provided by this natto purine metabolomics research, modifying the purine metabolic pathway could enhance its health-promoting value.

The composition and abundance of amino acids are vital indicators of nutritional quality in food and significantly influence taste perception [33]. Our study identified 45 amino acids, 26 of which exhibited differential accumulation between natto and soybeans. Notably, we did not observe elevated levels of sweet-tasting carbohydrates in natto; instead, certain sugars associated with flavor have decreased, including D-maltose (fold change = 0.018), maltotriose (fold change = 0.016), stachyose (fold change = 0.015), maltotetraose (fold change = 0.01), turanose (fold change = 0.007), lactulose (fold change = 0.007), and betadex (fold change = 0.0007). Moreover, natto contains higher concentrations of alkaloids. These metabolic constituents and discrepancies contribute to the distinctive flavor profile of natto. Therefore, in subsequent fermentation processes, the addition of corresponding sweeteners with prebiotic functions, such as D-allose, D-xylose, and D-tagatose, which possess sweetness comparable to sucrose and without caloric content, could be considered to enhance the flavor of natto. Similarly, natto contains functional sugars like D-xylose and D-tagatose, which augment its health-promoting properties. Furthermore, significant quantities of benzocaine and strictosidine were detected in natto. Benzocaine, a recognized anesthetic, and strictosidine, a key intermediate in monoterpene indole alkaloid biosynthesis, imply potential applications in medical anesthetics.

The KEGG enrichment analysis of the metabolite profile of natto demonstrated that the majority of metabolites were associated with three major metabolic pathways: isoflavone biosynthesis, purine metabolism, and the biosynthesis of tropane, piperidine, and pyridine alkaloids. This association establishes a theoretical framework for a deeper comprehension of the biochemical synthesis pathways of the primary metabolites in natto. As the final products of various biological processes and the material manifestation of gene expression, metabolites can potentially serve as accurate biomarkers of upstream biological events [27]. This research provides essential metabolomic data and opens new research avenues for further clarifying the biochemical mechanisms governing metabolite production and the physiological functions of fermented natto. Furthermore, it lays the groundwork for enhancing the health-promoting properties of natto through the optimization of fermentation techniques and modulation of metabolic pathways.

## Data Availability

The data presented in this study are available in the Supplementary Material.

## Supplementary Materials

The following supporting information can be downloaded at: https://www.mdpi.com/article/10.3390/metabo14120663/s1, Table S1: Metabolic signals detected in the soybeans and natto; Table S2: KEGG pathway; Figure S1: Metabolic pathway of isoflavonoid biosynthesis.
