# Supplementary figures and images for: Widely Targeted Metabolomics Revealed the Metabolic Basis of Physiological Function and Flavor of Natto
Source: Metabolites. 2024 Dec 1;14(12):663. doi: 10.3390/metabo14120663 (PMC11676936; doi:10.3390/metabo14120663)

### Figure S1

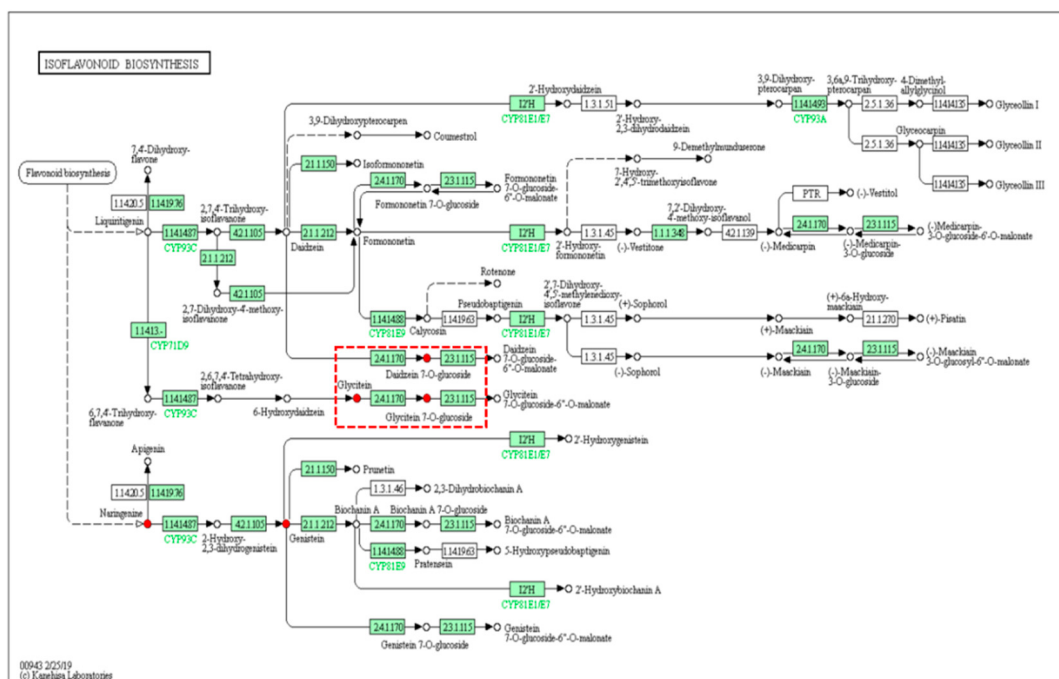

Figure S1. Metabolic pathway of isoflavonoid biosynthesis.

Supplement: Supplementary file 1 [file metabolites-14-00663-s001.zip › supplemental material.pdf]
